# An Atlas for *Schistosoma mansoni* Organs and Life-Cycle Stages Using Cell Type-Specific Markers and Confocal Microscopy

**DOI:** 10.1371/journal.pntd.0001009

**Published:** 2011-03-08

**Authors:** James J. Collins, Ryan S. King, Alexis Cogswell, David L. Williams, Phillip A. Newmark

**Affiliations:** 1 Howard Hughes Medical Institute, Department of Cell and Developmental Biology, Neuroscience Program, University of Illinois at Urbana-Champaign, Urbana, Illinois, United States of America; 2 Department of Immunology/Microbiology, Rush University Medical Center, Chicago, Illinois, United States of America; Biomedical Research Institute, United States of America

## Abstract

Schistosomiasis (bilharzia) is a tropical disease caused by trematode parasites (*Schistosoma*) that affects hundreds of millions of people in the developing world. Currently only a single drug (praziquantel) is available to treat this disease, highlighting the importance of developing new techniques to study *Schistosoma*. While molecular advances, including RNA interference and the availability of complete genome sequences for two *Schistosoma* species, will help to revolutionize studies of these animals, an array of tools for visualizing the consequences of experimental perturbations on tissue integrity and development needs to be made widely available. To this end, we screened a battery of commercially available stains, antibodies and fluorescently labeled lectins, many of which have not been described previously for analyzing schistosomes, for their ability to label various cell and tissue types in the cercarial stage of *S. mansoni*. This analysis uncovered more than 20 new markers that label most cercarial tissues, including the tegument, the musculature, the protonephridia, the secretory system and the nervous system. Using these markers we present a high-resolution visual depiction of cercarial anatomy. Examining the effectiveness of a subset of these markers in *S. mansoni* adults and miracidia, we demonstrate the value of these tools for labeling tissues in a variety of life-cycle stages. The methodologies described here will facilitate functional analyses aimed at understanding fundamental biological processes in these parasites.

## Author Summary

Schistosomes are parasitic flatworms that infect hundreds of millions of people worldwide. The development of genomic resources and recent application of functional genomic tools (e.g., global gene expression studies, inhibition of gene expression by RNA interference, and transgenesis) hold the promise of revolutionizing the study of schistosome biology. These advances necessitate the introduction of molecular markers for examining the consequences of manipulating schistosome genes. In this manuscript we report the use of several cell type-specific markers and confocal microscopy for visualizing various schistosome tissues in a variety of life-cycle stages. Our analysis provides an atlas of the major organ systems in three different life-cycle stages in these important parasites. The tools and methodologies reported here are widely available and can be readily adopted by researchers interested in more detailed studies of these organisms. We anticipate that these resources will be particularly useful for detailed phenotypic characterization following gene inhibition or over-expression studies.

## Introduction

Flatworms of the genus *Schistosoma* are parasites (Phylum Platyhelmithes) that currently infect over 200 million people worldwide [1]. Similar to other trematodes, *Schistosoma* have complex life cycles consisting of both free-living and parasitic forms [2]. Upon passage from a vertebrate, schistosome eggs that reach freshwater will hatch and produce miracidia, that swim by ciliary movement to locate and penetrate a suitable snail host. Following entry into the snail, miracidia undergo a dramatic developmental conversion, resulting in the production of primary and then secondary sporocysts, which have the capacity to generate thousands of cercariae. Cercariae are then liberated from the snail into freshwater where they can penetrate the epidermis of a vertebrate host. These animals (now called schistosomula) enter the host's circulatory system, eventually migrating to the liver where they feed on blood and develop to adulthood as either male or female worms. Separate sexed worms then pair, begin reproducing, and complete the life cycle by laying eggs that are passed via the urine or feces depending on the schistosome species.

Despite the daunting complexity of the schistosome life cycle, great strides have been made in developing modern tools to study these parasites. RNA interference has been used to disrupt gene function in eggs [3], [4], transforming cercariae [5], schistosomula [6], [7], [8], and adults [3], [9]. Such breakthroughs have opened the possibility for large-scale RNAi-based screens [8], [10]. Furthermore, transgenic approaches [11], and whole-mount in situ hybridization [15] techniques have been described, providing additional avenues to analyze gene function. These tools, combined with access to complete genome sequences for *S. mansoni* [16] and *S. japonicum* [17], will facilitate a greater understanding of these parasites, making this an exciting time for schistosome research. To fully realize the potential of these newly developed functional genomic tools, however, methods for analyzing changes in cell/tissue morphology following experimental perturbations will need to be developed. Furthermore, a collection of reagents to label distinct organ systems in a variety of life-cycle stages will facilitate a greater understanding of the complex developmental transitions these animals experience. One successful methodology for examining schistosome anatomy has been the combination of fluorescence microscopy with histological stains, such as carmine red or phalloidin. For example, carmine staining has been used to describe the reproductive anatomy of paired vs. unpaired adult females [18], [19] and phalloidin has been utilized to describe the musculature of a number of life-cycle stages [20], [21], [22]. Although these reagents will continue to be valuable tools, they are limited by their individual specificities; thus, there is a need to identify additional markers that will allow high-resolution analyses of distinct schistosome cell types and tissues.

Immunofluorescence microscopy has become an indispensable tool for detailed examination of tissue morphology during development and following experimental perturbation. Although species-specific antibodies are widely available for classic model organisms (e.g. *Drosophila* and mouse), only a limited set of these reagents have been generated for organisms with smaller research communities (e.g. *Schistosoma*). In emerging model systems such as planarians, a free-living relative of parasitic trematodes, systematic examination of cross-reactivity with commercially available antibodies from other species [23], [24] has been used to overcome the limited access to species-specific immunological resources. Such analyses identified markers for the planarian nervous system, stem cells, protonephridia, intestine, and reproductive system. A similar approach has been used to identify antibodies that label the schistosome nervous system (e.g. [25], [26], [27]); however, this approach has not yielded useful markers for other cell and tissue types. Lectins are proteins that recognize specific carbohydrate moieties, and when conjugated to reporter molecules (e.g. enzymes or fluorophores) can be useful for labeling specific vertebrate [27] and invertebrate [28], [29] tissues. Although some lectins have been used previously to label schistosome tissues [30], [31], [32], [33], [34], detailed descriptions of their labeling have not been reported using modern methodologies such as confocal microscopy.

Here we identify and characterize a collection of antibodies and lectins that label specific tissues of *S. mansoni* cercariae and use these stains to provide a detailed description of cercarial anatomy. We also examined both adults and miracidia and present a description of the protonephridial, reproductive and nervous systems in these stages using these tools. Together, these studies provide new tools and methods for studying these important parasites.

## Materials and Methods

### Obtaining free-living stages of *S. mansoni*

Schistosome-infected (Puerto Rican strain NMRI) Swiss-Webster mice were provided by the Schistosome Resource Center. *S. mansoni* eggs were obtained from mouse livers essentially as previously described [35]. To obtain miracidia, eggs were resuspended in artificial pond water (0.46 µM FeCl_3_ ⋅6 H_2_O, 220 µM CaCl_2_ ⋅2 H_2_O, 100 µM MgS0_4_ ⋅7 H_2_O, phosphate buffer [313 µM KH_2_PO_4_, 14 µM (NH_4_)_2_SO_4_] pH 7.2) and exposed to light either in a darkened side-arm flask [35] or a 24-well tissue culture plate [36]. *S. mansoni* cercariae were obtained from *Biomphalaria glabrata* snails (Schistosome Resource Center) by exposing snails to direct light at 28°C for ∼1–2 hours. *B. glabrata* snails were maintained in artificial pond water and fed Layer Crumbles (chicken feed) (Rural King, Mattoon, IL). In adherence to the Animal Welfare Act and the Public Health Service Policy on Humane Care and Use of Laboratory Animals, all experiments with and care of vertebrate animals were performed in accordance with protocols approved by the Institutional Animal Care and Use Committee (IACUC) of the University of Illinois at Urbana-Champaign (protocol approval number 10035).

### Fixation and staining of miracidia and cercariae

For fixation of miracidia and cercariae, an 8% formaldehyde solution was prepared by diluting 36% formaldehyde (EMD Chemical, Darmstadt, Germany) in artificial pond water. This solution was added to an equal volume of miracidia or cercariae (also in artificial pond water), agitated vigorously, and incubated for 20–25 m, except for staining with anti-synapsin where samples were fixed for 2 h. Typical volumes for fixation were >20 ml. Fixed animals either settled by gravity or were pelleted by a brief spin (∼5–10 s) in an Eppendorf 5810R centrifuge (Hamburg, Germany) with brake settings on 0 or 1. Following fixation, samples were rinsed in PBSTx (PBS + 0.3% Triton X-100). For subsequent steps, we found two convenient methods for specimen handing. First, samples were aliquoted to a 96-well microtiter plates and allowed to settle by gravity between liquid exchange steps. Alternatively, samples could be processed in 1.7 ml microcentifuge tubes and liquid exchanges could be performed either after gravity settling of the samples or following a brief spin (<5 sec.) in a benchtop microfuge.

The staining with some reagents was significantly improved by a brief Proteinase K digestion. For this treatment, samples were incubated in Proteinase K solution (2 µg/mL (Invitrogen, Carlsbad, CA), 0.5% SDS, in 1X PBSTx) for 5 min, allowed to settle by gravity, and post-fixed in 4% formaldehyde in PBSTx for 10 min. Since staining with the anti-synapsin antibody was sensitive to permeabilization with Proteinase K treatment, samples were permeabilized for 20 min in 1x PBS + 1% SDS. Permeabilization techniques for specific stainings are listed in Table S1.

Following permeabilization, animals were rinsed in PBSTx, incubated in blocking solution (5% horse serum, 0.45% fish elatin, 0.3% Triton X-100, 0.05% Tween 20 in 1x PBS) for ∼2 hours and then incubated in primary antibody diluted in blocking solution (concentrations indicated in Table S1) for >6 h at 4°C. Following >2 h of washing in PBSTx, samples were incubated in goat anti-mouse Alexa fluor 488 (Invitrogen, Carlsbad, Ca; 1∶400–1∶500) for 6 hours to overnight at 4°C. Occasionally, DAPI (1 µg/mL final in PBSTx) and phalloidin conjugated to either rhodamine or Alexa fluor 633 (66 nM final in PBSTx) were added during secondary antibody incubations or wash steps. As an alternative, we experimented with tyramide signal amplification (TSA) to detect antibody staining. For TSA detection, a goat anti-mouse HRP-conjugated secondary antibody (Invitrogen, Carlsbad, CA) was diluted (1∶100) in blocking solution and incubated with samples for 2 hr at room temperature. Samples were washed for 2 hrs in PBSTx and incubated in Amplification Diluent containing fluorescein tyramide (1∶50; TSA-Plus, Perkin Elmer, Waltham, MA). For anti-synapsin staining, TSA resulted in superior signal-to-noise ratios when compared to detection with Alexa Fluor-conjugated secondary antibodies (data not shown). Although this methodology was optimized for anti-synapsin staining, it is anticipated that similar results would be obtained with other murine antibodies.

For lectin staining, animals were fixed and blocked as described above and then incubated overnight at 4°C with a fluorophore-conjugated lectin diluted 1∶500 in blocking solution. Lectins were obtained from Vector Laboratories (Burlingame, CA) and diluted from 2 mg/mL stocks. Samples were washed in PBSTx and stained with a combination of DAPI and Alexa fluor 633 phalloidin.

### Fixation and staining of adult *S. mansoni*

Adult *S. mansoni* perfused from mice [35] were fixed for 30 minutes at room temperature in 4% formaldehyde diluted in PBSTx. Following brief rinses in 1x PBSTx samples were dehydrated through a methanol/PBSTx series (25%, 50%, 75%, and 100% MeOH) and stored at −20°C until use. Samples were rehydrated by incubation in 1∶1 MeOH:PBSTx followed by incubation in PBSTx. Rehydrated samples were treated with Proteinase K (2 µg/mL) for 10 minutes at room temperature and then post-fixed for 10 minutes in 4% formaldehyde in PBSTx. Samples were processed for immunohistochemistry and lectin staining similar to cercariae and miracidia. For staining with phalloidin, methanol dehydration and Proteinase K treatments were omitted.

### Imaging

Samples were mounted in Vectashield (Vector Laboratories, Burlingame, CA) or a mixture of Vectashield and 80% glycerol and imaged on a Zeiss LSM 710 confocal microscope (Carl Zeiss, Germany) (Plan-Apochromat 63x/1.4 Oil DIC objective). Alexa 488/FITC, Alexa 568/Rhodamine, and Alexa 633 flours were excited with 488 nm, 561 nm, and 633 nm lasers, respectively. Images were processed either using Zen 2009 (Carl Zeiss, Germany), ImageJ [37], or Imaris (Bitplane AG Zurich, Switzerland). Movies were made using iMovie '09 (Apple, Cupertino, CA).

## Results

To elucidate cell- and tissue-specific markers useful for anatomical analyses of *Schistosoma*, we examined a panel of antibodies, lectins and histological stains in *S. mansoni* cercariae. We chose cercariae over other stages because these animals are easily maintained in the laboratory and can be obtained in large numbers, facilitating the optimization of fixation and permeabilization conditions. Furthermore, since the cercariae possess many tissues present in other life-cycle stages (e.g. protonephridial and nervous systems), we predicted that cercarial markers would have utility in other stages. Below we report the results of these studies with our observations reported by organ system.

### Surface structures and musculature

**Figure 1:**
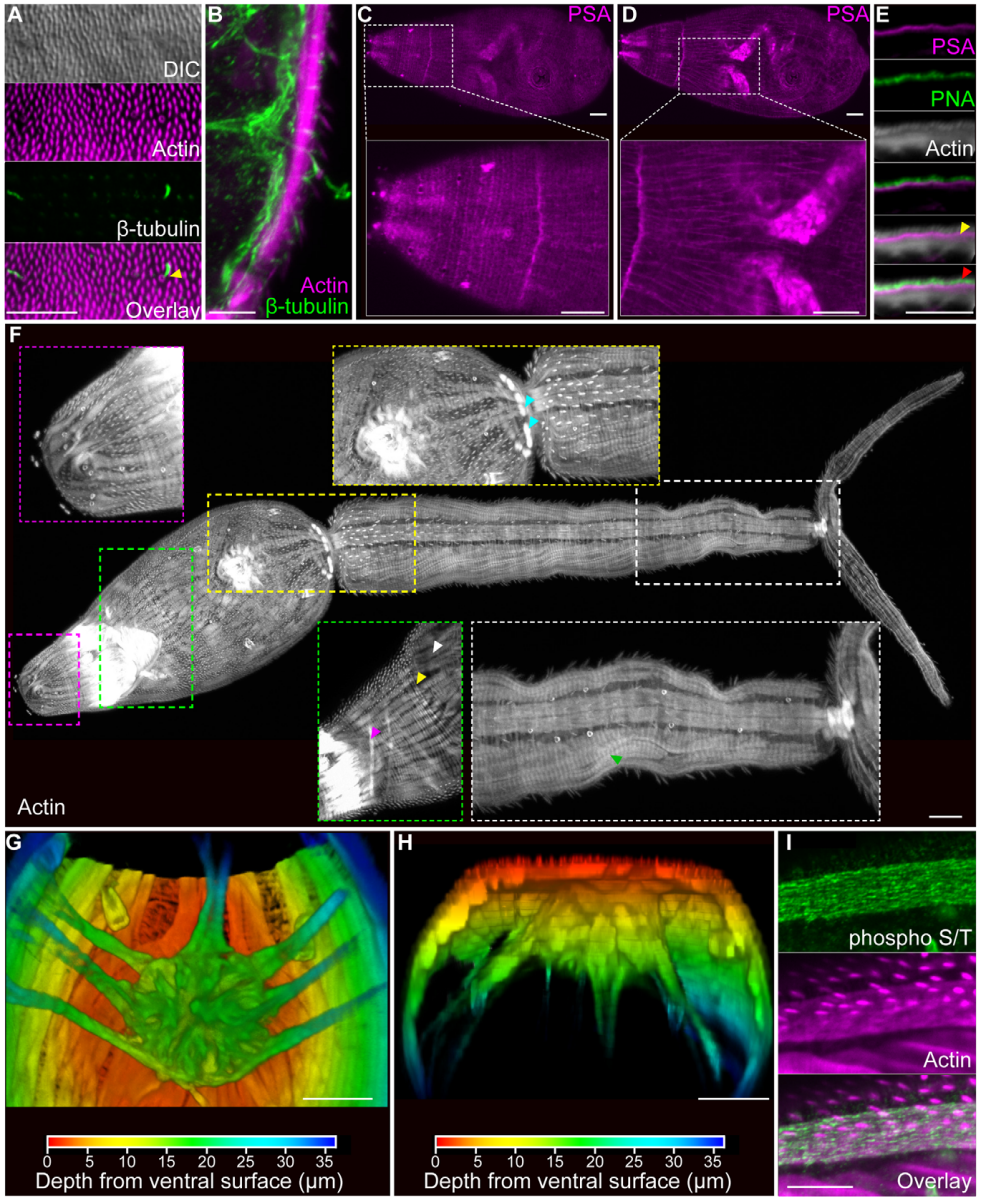
Superficial structures and musculature of cercariae. (A and B) Actin-rich spines and sensory cilia. (A) Anterior region of the head as seen by Differential Interference Contrast (DIC) optics, phalloidin staining to visualize actin, and immunofluorescence with an anti-β-tubulin antibody to label cilia. Images are maximum confocal projections. Bottom, overlay showing distribution of actin and β-tubulin. Arrow indicates an actin-rich channel though which a sensory cilium projects. (B) Maximum confocal projection of a cross section though the tail showing a sensory cilium (green) crossing the musculature (magenta) to project to the outside (right). (C and D) Single confocal sections depicting staining with lectin PSA that labels the basement membrane below the tegument. Panels C and D represent distinct confocal sections of the same animal. (E) Single confocal section through the head showing staining with lectins PSA and PNA and with phalloidin to visualize actin. PSA labels the basement membrane between the actin-rich surface spines and the muscle layer (yellow arrow). PNA marks a layer of material at the level of the actin spines which may represent the tegument or the associated glycocalyx (red arrow). (F) Phalloidin staining in various anatomical regions. Magenta box, actin spines and anterior sensory structures. Green box, longitudinal (white arrowhead), circular (yellow arrowhead), and diagonal muscle fibers (magenta arrowhead). Yellow box, acetabulum and the interface between the head and the tail. Note the intense radially symmetric spheres of phalloidin staining (cyan arrowhead); because of the proximity of these structures to longitudinal muscles in the head and tail, we suggest this staining may represent sites of muscle attachment. White box, longitudinal and helical muscles (green arrowhead) of the tail. Whole cercaria image represents a maximum projection derived from tiled stacks. Insets are magnified views of the indicated regions. All images are maximum projections generated from a Z-stack though an entire animal, except for the green inset that was derived from a subset of optical sections. (G and H) Depth projections showing phalloidin staining of the acetabulum and associated musculature. Scale shown below indicates the color-coding of distances from the ventral surface (i.e. the colors transition from red (ventral) to blue (dorsal) moving deeper into the animal). Panel G represents a dorsal view whereas panel H depicts a transverse section with the ventral surface towards the top. (I) Immunofluorescence with an anti-phospho S/T antibody that labels the longitudinal muscles of the tail. Scale bars, 10 µm. Anterior faces up in panel A and to the left in panels C, D, and F.

Cercaria are covered by a sugar-rich layer, called the glycocalyx, that sits upon the tegument, a modified syncytial epidermis characterized by a trilaminate membrane system [2], [38], [39]. In *S. mansoni* cercariae spines that face posterior and cover nearly the entire surface of the animal are prominent features of the tegument [20], [38]. Similar to previous reports [20], we find that these spines can be labeled with phalloidin (Figure 1A), consistent with these spines being rich in filamentous actin. Because the goal of this life-cycle stage is to locate and penetrate a suitable host, cercariae also possess a variety of ciliated sensory papillae [38]. We observed ciliated regions of unsheathed papillae scattered along the entire length of the animal by staining with antibodies specific to β-tubulin (Figure 1A, B) and acetylated α-tubulin (Table S1). Additionally, the openings of these ciliated papillae could be visualized as phalloidin-stained discs (Figure 1 A, B) that encircle cilia projecting through the tegument(Figure 1A, B).

The cercarial basement membrane serves as the interface for the attachment of the exterior tegument and the underlying body wall musculature. In cercariae this structure stained with a variety of lectins such as *Pisum sativum* lectin (PSA) (Figure 1C, D, E and Table S1). Double labeling with PSA and Peanut lectin agglutinin (PNA), revealed that PNA marked a layer of material outside the basement membrane at the level of the actin-rich spines embedded in the tegument. This PNA labeling could represent the tegument itself or the sugar-rich glycocalyx coating the outside of the cercarial tegument [38].

Similar to free-living flatworms, the body wall musculature of the cercariae consists of an external layer of circular muscles situated above longitudinal muscles that are interweaved with diagonal fibers [39]. Phalloidin stained all three muscle layers within the animal (Figure 1F and Movie S1) as well as other structures such as the acetabulum (ventral sucker) (Figure 1G and H), mouth, esophagus, and flame cells of the protonephridial system (described below). Since a previous study used phalloidin staining to describe the cercarial musculature [20], we refer readers to this reference for a detailed description of phalloidin staining. In addition to phalloidin staining, we were able to visualize the longitudinal muscles of the tail with an antibody that recognizes phosphorylated serine and threonine (anti-phospho S/T) residues (Figure 1I). Since we failed to observe anti-phospho S/T labeling in other cercarial muscle fibers, it is possible that these muscles are physiologically distinct from other muscles in the animal.

### Cercarial glands

**Figure 2:**
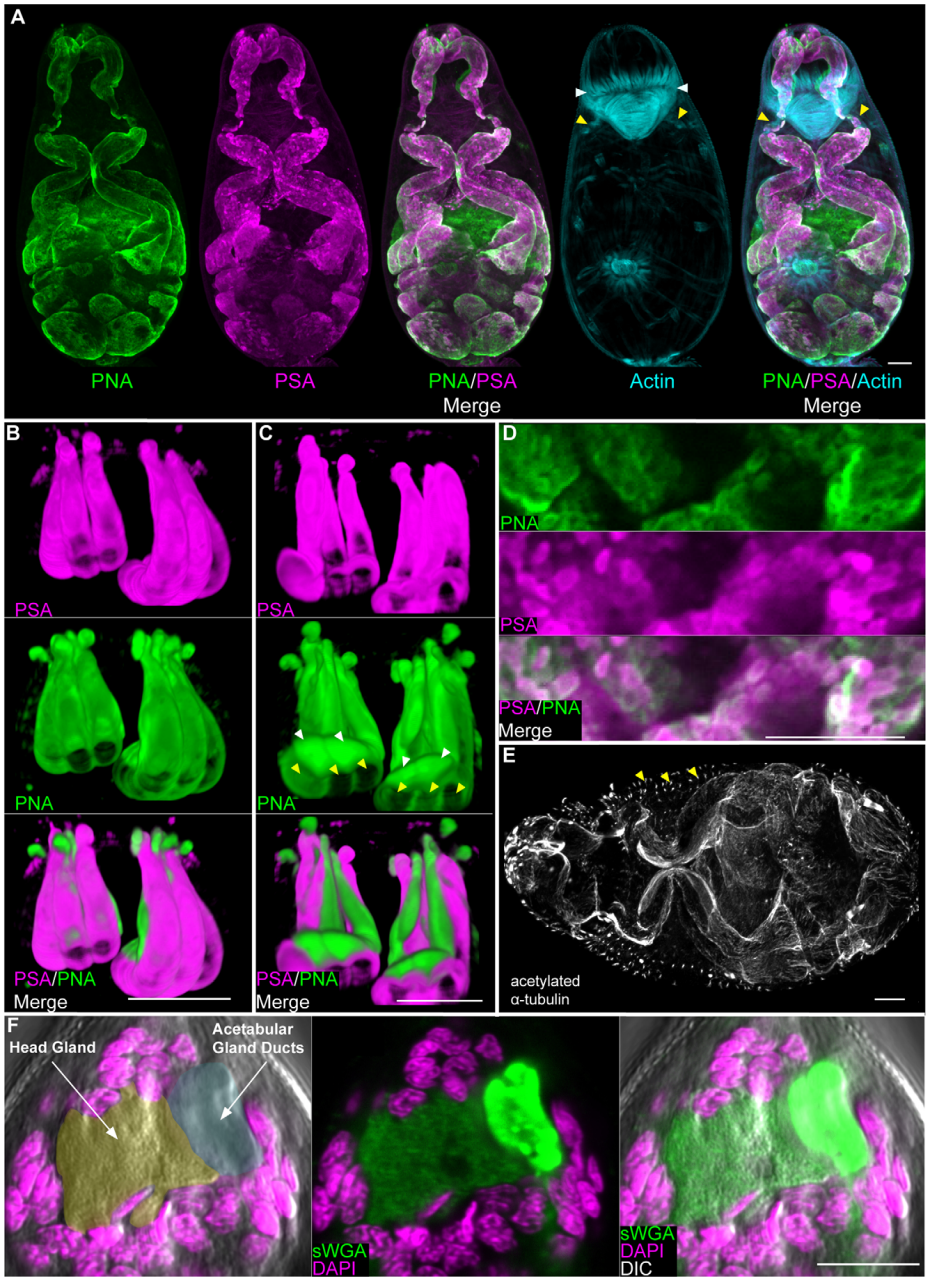
The secretory glands of cercariae. (A) Maximum confocal projections showing labeling of the entire pre- and post- acetabular gland system with lectins PNA and PSA. Yellow arrowheads indicate musculature surrounding the anterior ducts of the acetabular glands and white arrowheads indicate the muscle cone. (B and C) 3D-renderings showing magnified views of the acetabular gland ducts anterior to the muscle cone. The six post-acetabular gland ducts (yellow arrowheads) labeled with both PNA and PSA. PNA labeled the four pre-acetabular ducts (white arrowheads) whereas PSA did not. Panel B represents a ventral view; panel C represents a dorsal view. (D) Secretory globules within the post-acetabular glands labeled with PNA and PSA. (E) Immunofluorescence with an anti-acetylated α-tubulin antibody showing the microtubule-rich periphery of the pre- and post- acetabular glands. Arrowheads indicate putative sensory papillae. (F) sWGA labels the head gland. Left, DIC and DAPI staining. Head gland is pseudocolored orange and acetabular gland ducts are pseudocolored cyan. Center and right most panels show staining with sWGA. Scale bars, 10 µm. Anterior faces up in panels A, B, C and F and to the left in panel E.

The five pairs of acetabular glands (three pairs of post-acetabular and two pairs of pre-acetabular) are prominent features of the cercarial anatomy, occupying nearly two-thirds of the volume of the cercarial head [38], [40]. The fundi of these unicellular glands fill the body cavity beneath the acetabulum and project anteriorly through the muscle cone, terminating at the anterior apex of the animal. Although the precise function of these glands has yet to be resolved, the secretion of proteinases rom the pre-acetabular glands [41] and mucus-like substances from the post-acetabular glands [42] suggests roles in host penetration and adhesion. Consistent with previous observations of live cercariae [34], a number of fluorescently labeled lectins stained the acetabular glands (Table S1, Figure 2A, Movie S2), both at their base and along their projections. While most of these lectins labeled the post-acetabular fundi, a subset stained both the pre- and post-acetabular glands. Among these was PNA, which labeled the pre- and post-acetabular glands as well as all ten of their projections (Figure 2A, B, C). Co-labeling with lectins (e.g. PSA or PNA) and phalloidin revealed a ring of musculature surrounding the acetabular ducts just posterior to the muscle cone (Figure 2A and Movie S2). Given the position of these muscles, they may act to initiate contractile forces that expel secretions from the ducts or to control secretion release.

Double labeling with lectins PSA and PNA showed that these individual lectins label unique sub-cellular regions of the post-acetabular glands (Figure 2A). This was most obvious in the ducts, in which PNA staining was concentrated towards the periphery and PSA staining was detected throughout the duct (Movie S2). Furthermore, secretory globules [38], [40] scattered throughout the posterior regions of the glands were labeled to varying extents with either PSA and/or PNA (Figure 2D). This heterogeneous labeling might reflect individual differences in the content of these secretory globules. Ultrastructural studies describe a microtubule network surrounding the entire acetabular gland system [38], [40]. Consistent with these studies we were able to label the fundi and ducts of these glands with anti-acetylated α-tubulin (Figure 2E).

In addition to the acetabular glands, cercariae possess a unicellular gland at the anterior end called the "head gland". This gland has been suggested to provide membrane material required during the cercariae-schistosomula transition following host penetration [43]. Similar to other cercarial glands, the head gland was readily visualized with a variety of lectins (Table S1, Figure 2F).

### The protonephridial system

**Figure 3:**
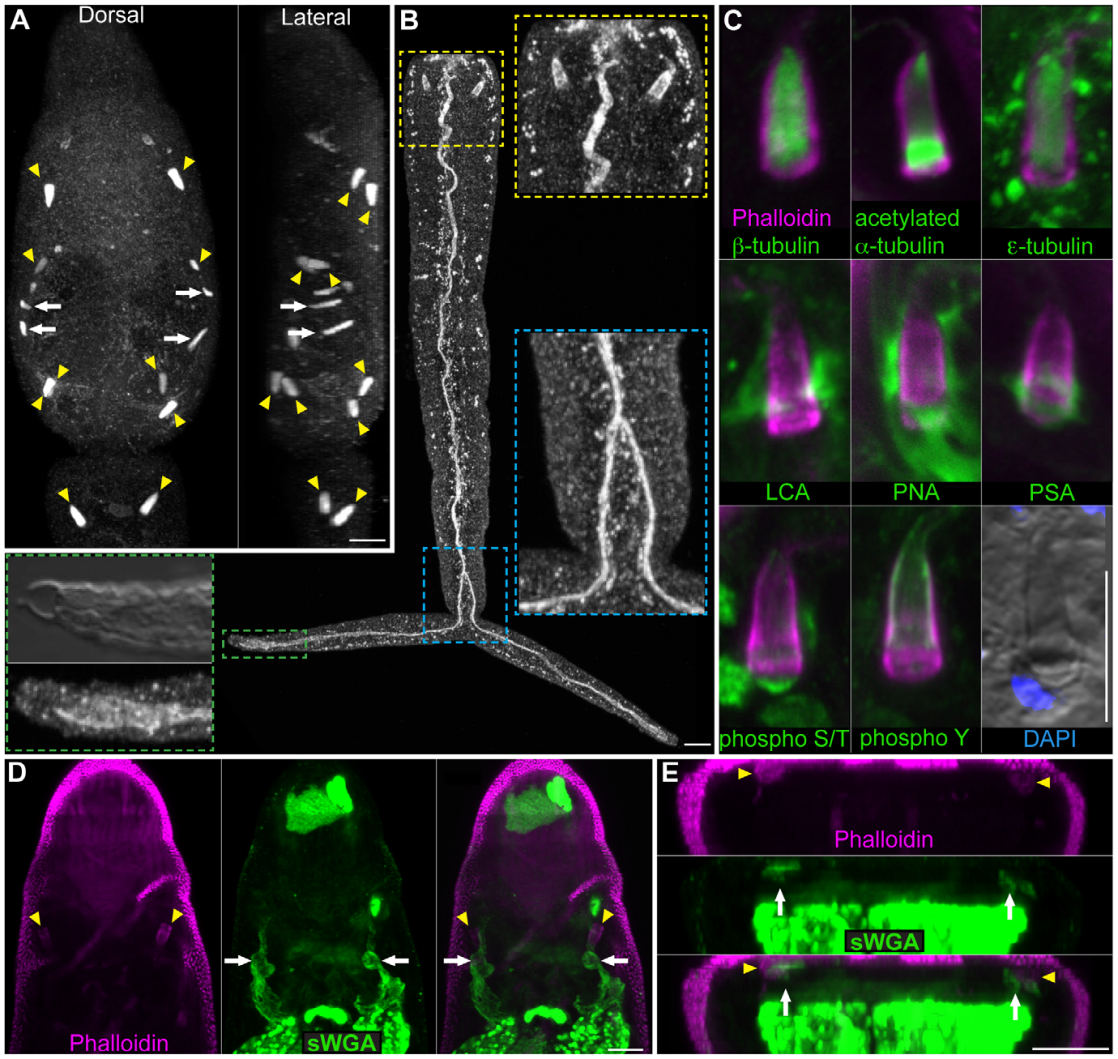
The protonephridial system of cercariae. (A) Immunofluorescence with an anti-β-tubulin antibody to label the ciliated tufts of the flame cells (yellow arrowheads) and ciliated regions of the protonephridial tubules (white arrows) within the head and tail. Cercariae typically have 10 flame cells, an anterior dorsal pair, a mid-head ventral pair, posterior head dorsal and ventral pairs, and a pair at the anterior tail. Dorsal view is shown to the left and a lateral view is shown to the right. (B) Immunofluorescence with an anti-phospho Y antibody that labels the barrel of the flame cells and the excretory duct. Inserts are magnified views of the indicated regions. Yellow box, flame cells of the tail. Blue box, the protonephridial tubule splits anterior to the point of tail bifurcation. Green box, the protonephridial tubule extends to the nephridiopore at the tip of the tail. Mid-level DIC optical section showing nephridiopore and tegumental structure at tip of tail. (C) Numerous reagents utilized in this study labeled portions of the flame cells providing useful tools for analyzing flame cell morphology. The ciliated tuft stained with various anti-tubulin antibodies (top row). Several lectins showed different staining patterns of the extracellular matrix surrounding the flame cell (middle row). Phospho-specific antibodies (bottom row) also labeled the flame cells: anti-phospho S/T labeled the ciliary rootlet and anti-phosopho Y labeled the flame cell barrel. Differential interference contrast optics also permit observation of flame cell morphology. (D and E) Portions of the protonephridial tubules in the head label with lectin sWGA. (D) Maximum intensity projection of dorsal focal planes showing sWGA labeling of protonephridial tubules (white arrows) leading from the anterior flame cell pair (yellow arrowheads). (E) Cross-section of a maximum intensity projection from animal in D showing the sWGA-labeled protonephridial tubules (arrows) positioned dorsal to the sWGA-labeled acetabular ducts. Abbreviations: *Lens culinaris* agglutinin (LCA), peanut agglutinin (PNA), *Pisum sativum* agglutinin (PSA), succinylated wheat germ agglutinin (sWGA). Scale bars, 10 µm. Anterior faces up in all panels.

After being shed from their snail host and before locating and penetrating a mammal, cercariae spend a substantial portion of their short lives in freshwater. Cercaria may regulate water balance by controlling permeability through their outer surface (e.g. the tegument and/or glycocalyx) and by excretion of excess fluid by a network of osmoregulatory tubules called protonephridia [44], [45]. Proximally the protonephridia begin as a heavily ciliated cell called a flame cell. Staining of cercariae with antibodies that recognize various tubulin isoforms and modifications showed labeling of the ciliary tufts of flame cells (Figure 3A and C). Anti-tubulin antibodies also labeled several other structures, including portions of the nervous system (see below); proteinase K treatment often abolished this labeling, leaving predominantly protonephridial labeling (Table S1). For example, staining with the anti-β tubulin antibody following Proteinase K treatment provided robust and specific labeling of the flame cells (Figure 3A arrowheads) and ciliated secondary protonephridial tubules (Figure 3A arrows), allowing for easy identification of these cells even by epifluorescence. In contrast to a previous report stating that cercariae have six pairs of flame cells [38], we find that cercariae typically have 5 pairs of flame cells: an anterior-body dorsal pair, a mid-body ventral pair, posterior-body dorsal and ventral pairs, and a pair in the anterior tail (Figure 3A) similar to that described by Skelly and Shoemaker [46]. The base of the flame cell contains a region of tightly packed ciliary rootlets, which label strongly with an anti-phospho S/T antibody, and a nucleus, which is readily identified by DIC microscopy or DAPI staining (Figure 3C). The ciliary tuft of the flame cells sits in a barrel or basket-like structure formed by interdigitation between the flame cell and first tubule cell [38] and labels strongly with an anti-phospho tyrosine (anti-phospho Y) antibody (Figure 3B and 3C). In addition to labeling the flame cell and first tubule cell, anti-phospho Y also strongly labels the protonephridial tubule extending from the bladder to the nephridiopores located at the tips of the tail furci (Figure 3B). The protonephridial tubule splits anterior to the bifurcation of the tail (Figure 3B; blue inset). Although flame cells in the head were labeled by the anti-phospho Y antibody, it was unclear whether the protonephridial tubules were also labeled, since this antibody marked a variety of structures in the head (Table S1 and Movie S3).

Both the flame cells and first tubule cells contain an actin-rich network that is easily visualized using fluorescently labeled phalloidin (Figure 3C). Interestingly, several fluorescently labeled lectins showed differential staining of the extracellular matrix (ECM) surrounding the flame cells (Figure 3C). *Lens culinaris* agglutinin (LCA) labeled the ECM surrounding the first tubule cell down to the level where this cell interdigitates with the flame cell (Figure 3C). PNA showed labeling of the entire ECM surrounding the first tubule cell and flame cell. PSA labeling was restricted to the ECM surrounding the junction between the first tubule cell and flame cell. It has long been appreciated from ultrastructural observations that platyhelminth flame cells are surrounded by a significant amount of ECM [44]; our findings indicate surprising complexity in the composition of this material. We also observed lectin staining of portions of the protonephridial ducts in the cercarial head (Figure 3D and E and Table S1).

### The nervous system

**Figure 4:**
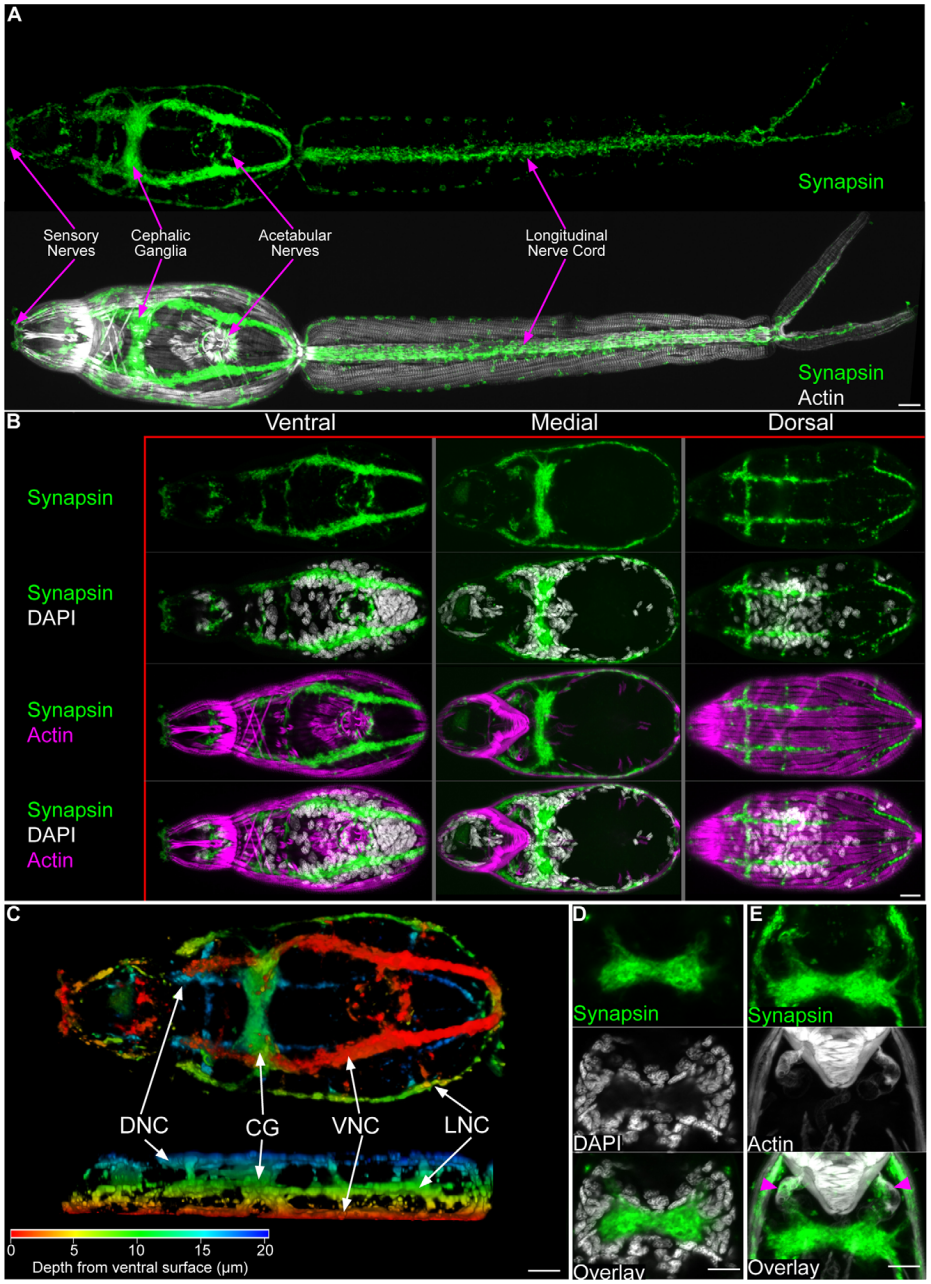
The central nervous system of cercariae. (A) Immunofluorescence with an anti-synapsin antibody that labels the cephalic ganglia and many peripheral neural structures. Below, synapsin labeling is shown together with phalloidin staining. (B) Maximum confocal projections generated from ventral, medial, or dorsal focal planes. Anatomical regions are given above and staining reagents are listed to the left. (C) Depth projection showing synapsin staining in the CNS. Ventral view is shown above and a lateral view is shown below. Scale shown below indicates color-coding of distances from the ventral surface. Abbreviations: Dorsal nerve cords (DNC), cephalic ganglia (CG), ventral nerve cords (VNC), lateral nerve cords (LNC). For the sake of simplicity we have not distinguished anteriorly projecting cords from dorsally projecting cords. (D) Single confocal section though the cephalic ganglia, showing the central neuropil surrounded by neuronal nuclei. (E) Confocal projection showing innervation between the cephalic ganglia and the musculature surrounding the anterior ducts of the acetabular glands (magenta arrowheads). Scale bars, 10 µm. Anterior faces left in panels A, B, and C and up in panels D and E.

The stereotypical flatworm central nervous system consists of paired cephalic ganglia that connect to nerve cords which extend through the body longitudinally [39], [47]. These nerve cords are often connected by transverse commissures, giving the flatworm nervous system a "ladder" or "orthogonal" appearance. Antibodies that recognize various neurochemical signaling molecules (e.g. neuropeptides) have been invaluable reagents for describing the neural anatomy of a variety of free-living and parasitic flatworms [47]. However, since the neurosignaling molecules recognized by these antibodies are often expressed in restricted cell types in flatworms [48], we explored additional markers that recognize features found more generally in neurons. A monoclonal antibody raised against the *D. melanogaster* Synapsin-1 protein, that is concentrated in nerve terminals of the fly [49], has been used to label the neuropil of the cephalic ganglia, the nerve cords, the transverse commissures, and the sub-muscular plexus of the planarian *S. mediterranea* [24]. We find this antibody labels similar structures in the central nervous system of cercariae. Specifically, anti-synapsin staining allowed visualization of the neuropil of the cephalic ganglia (Figure 4A, B, C, D and Movie S4), the six-pairs of longitudinal nerve cords (Figure 4A, B, C), and transverse commissures in the head (Figure 4A, B, C). Additionally, anti-synapsin labeled anterior sensory structures, nerves surrounding the acetabulum, and a pair of nerve cords below the longitudinal muscles of the tail. Of particular interest, we observed projections from the cephalic ganglia innervating muscles surrounding the ducts of the acetabular glands (Figure 4E and Movie S4). This innervation suggests that signals directly from the cephalic ganglia may be involved in controlling secretion from the acetabular glands during penetration.

**Figure 5:**
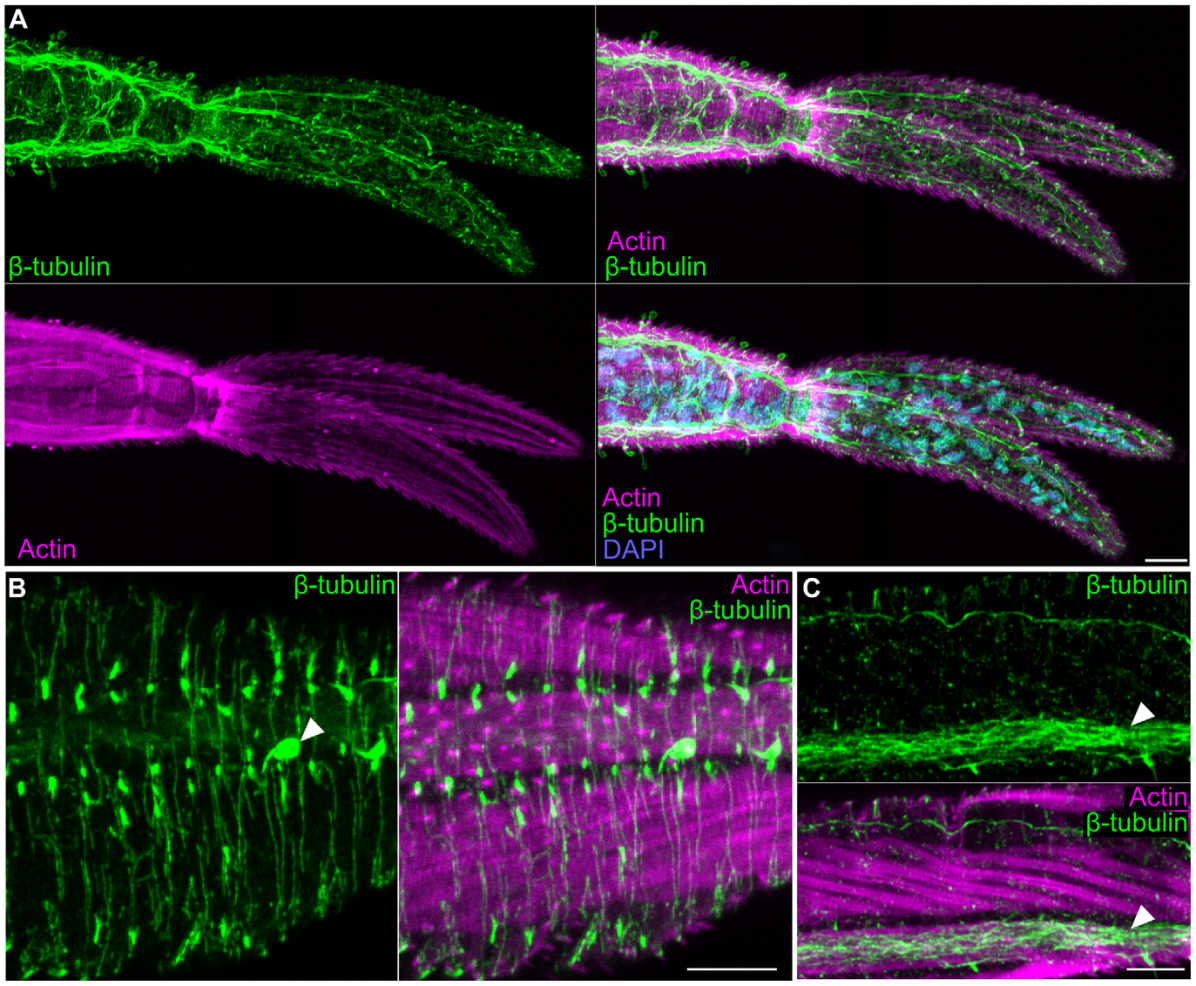
The nervous system of the cercarial tail. (A) Extensive neural projections within the tail visualized by β-tubulin immunostaining. Overlay with phalloidin and DAPI show the position of the nerves relative to the tail musculature and nuclei, respectively. (B) Superficial neural projections (green) laying outside muscle layer (magenta). Arrowhead indicates a sensory papilla. (C) Longitudinal nerve cord (white arrowhead) running along a longitudinal muscle within the tail. Scale bars, 10 µm. Anterior faces left in all panels.

In addition to anti-synapsin staining, we found that anti-β-tubulin was useful for detecting the nervous system within the tail (Figure 5A, B, C). However, unlike anti-synapsin that labels synaptic regions, anti-β-tubulin stained microtubule-rich neural projections throughout the tail. These fine projections could be observed transversely projecting through the interior of the tail (Figure 5A), superficially between the tegument and the muscles (Figure 5B), and within a pair of cords beneath the longitudinal muscle fibers (Figure 5C).

### Reagents useful for analyzing cercaria anatomy can also be used in adults and miracidia

**Figure 6:**
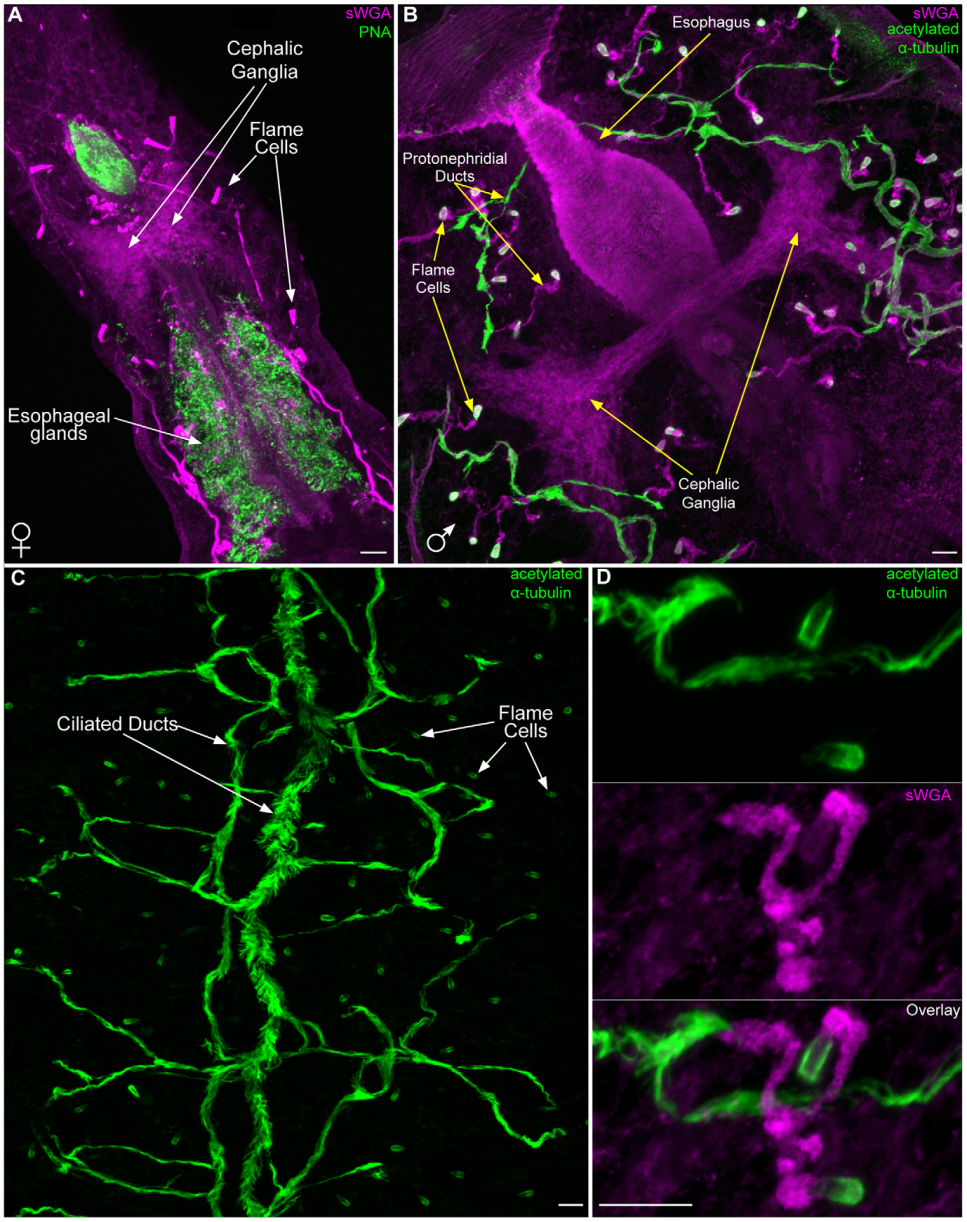
Labeling of non-reproductive tissues in adult *S. mansoni.* (A) Lectin PNA staining of the esophageal gland cells. Lectin sWGA labeling of cephalic ganglia and protonephridial ducts in a female. Image is a maximum intensity projection. (B) Lectin sWGA labeling of cephalic ganglia and non-ciliated protonephridial ducts of a male. Also shown is anti-acetylated α-tubulin staining the ciliated regions of the protonephridial system. Image is a maximum intensity projection. (C) Maximum intensity projection depicting anti-acetylated α-tubulin staining of the ciliated regions of the male protonephridial system, including the flame cells and the collecting ducts. Note that many secondary and tertiary ciliated ducts funnel into one of the two main collecting ducts that run along the longitudinal axis. (D) Maximum intensity projection of a protonephridial unit labeled with anti-acetylated α-tubulin and lectin sWGA. Anti-acetylated α-tubulin marks the ciliated flame cells and ducts, whereas sWGA labels non-ciliated tubules. Scale bars, 10 µm. Anterior faces to the upper left in panels A and B.

Following host penetration, *S. mansoni* cercariae (now schistosomula) enter the circulatory system and develop within the lungs before ultimately residing in the vessels of the hepatic portal system. Because cercariae are essentially the template from which the adult animals develop, we examined markers particularly useful for labeling cercariae (Lectins sWGA and PNA and the anti-acetylated α-tubulin antibody) in adult *S. mansoni*. Consistent with PNA labeling of secretory glands in cercariae (Figure 2A), we observed intense PNA staining of secretory glands surrounding the esophagus in the anterior of male and female worms (Table S1 and Figure 6A). Also similar to cercariae (Table S1), we observed sWGA labeling in the neuropil of the cephalic ganglia (Figure 6A, B) and the extracellular matrix/basement membrane of both sexes. Although adult schistosomes likely rely on the host for regulating fluid balance [44], these animals possess an extensive protonephridial system that consists of flame cells and an elaborate system of ciliated and unciliated ducts [50]. Like cercariae, the ciliated flame cells were detected with anti-acetylated α-tubulin and the ECM of the unciliated ducts stained with sWGA (Figure 6A–D, and Movie S5). Furthermore, anti-acetylated α-tubulin labeled the extensive network of ciliated collecting ducts (Figure 6B–D). Double labeling with sWGA and anti-acetylated α-tubulin allowed for staining of the complete protonephridial system (Figure 6B and D, and Movie S5).

**Figure 7:**
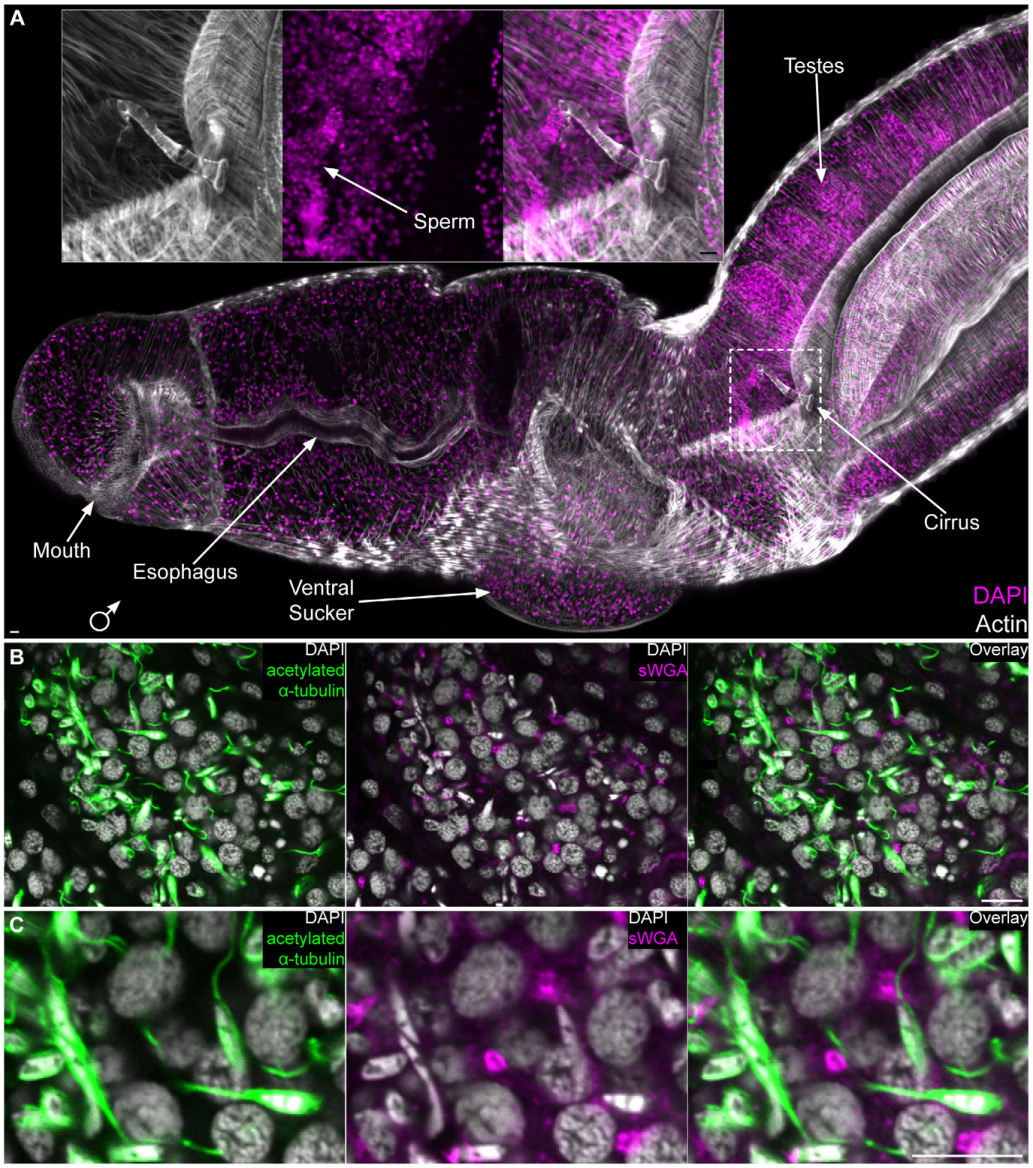
The male reproductive system. (A) Staining with DAPI and phalloidin showing the male head and various parts of the male reproductive system. Inset, magnified view of male cirrus. Image represents a maximum intensity projection derived from tiled stacks. Anterior to left, dorsal towards top. (B) Single confocal plane showing a testes lobe stained with anti-acetylated α-tubulin, sWGA and DAPI. DAPI staining shows the nuclear morphology of cells in different stages of spermatogenesis and anti-acetylated α-tubulin (green) stains the flagella and cortical microtubules of sperm. sWGA stains tubular structures that could represent cytoplasmic bridges between cells in mitotic and/or meiotic stages of spermatogenesis. (C) Magnified view of panel B. Scale Bars, 10 µm.

**Figure 8:**
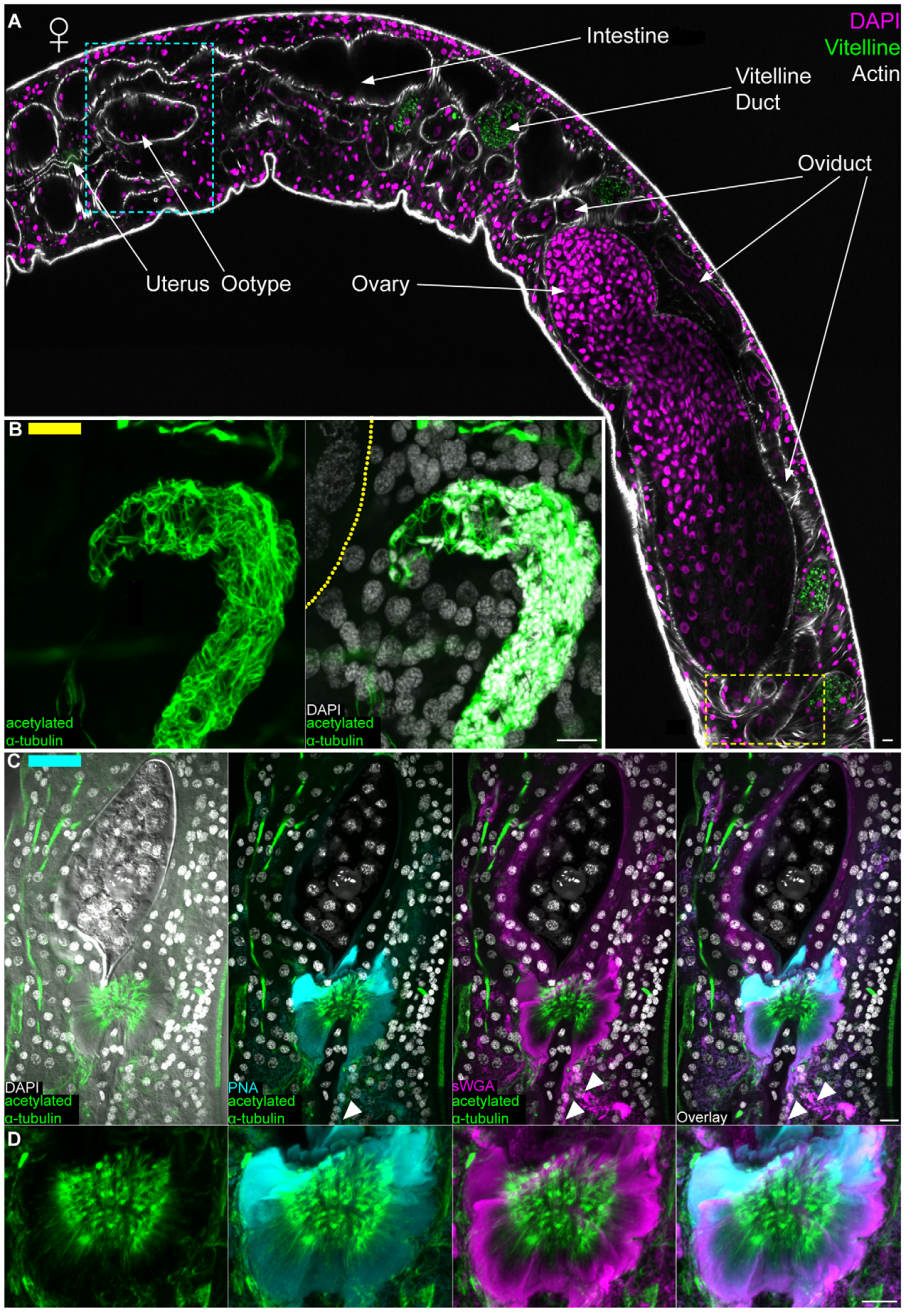
The female reproductive system. (A) Top, female labeled with DAPI and phalloidin to show various regions of the female reproductive system. Autofluorescence from the vitelline cells is shown in green; this autofluorescence served as a useful marker for the vitelline duct and its contents. Image represents tiled images of confocal sections from a single animal. Yellow and cyan boxes correspond to the regions of the seminal receptacle and the ootype/Mehlis' gland complex, respectively. These boxes are color coded to indicate the relative positions of the structures shown in either panels B (yellow bar in upper left) or C (cyan bar in upper left). (B) Left, anti-acetylated α-tubulin labeling microtubules of sperm present in the seminal receptacle. Right, large nuclei of oocytes in the ovary can be seen stained with DAPI to the left. Dashed line represents the position of the of muscle layer surrounding the ovary. Images are from mid-level maximum intensity projections. (C) Maximum intensity projections showing staining of the ootype (top, shown surrounding an egg) with sWGA and Mehlis' gland with PNA, sWGA and acetylated α-tubulin (bottom). Arrowheads indicate cytoplasmic projections of Mehlis' gland cells. (D) Magnified view of Mehlis' gland from panel C. Scale Bars, 10 µm. Anterior faces left in panel A and up in panels B, C and D.

We also found sWGA, PNA and anti-acetylated α-tubulin to be useful for visualizing the male and female reproductive organs. The *S. mansoni* male reproductive system consists of four to nine testes [2], [51]; the sperm they produce are passed to the female via a cirrus at the anterior end of the gynecophoral canal (Figure 7A). Cortical microtubules surrounding the sperm head and within the sperm flagella were visualized within testes lobes by staining with anti-acetylated α-tubulin (Figure 7B, C). Additionally, we observed sWGA labeling tubular (doughnut shaped) structures between a subset of nuclei in the testes (Figure 7B, C). Because developing germ cells in other animals often remain attached to one another by cytoplasmic bridges (e.g. ring canals [52]), we speculate that these structures could represent canals connecting the cytoplasm of germ cells undergoing spermatogenesis. Spermatozoa passed to a female migrate up the female reproductive tract and are stored in the seminal receptacle located posterior to the ovary; tightly packed sperm were observed in this receptacle by staining with DAPI and anti-acetylated α-tubulin (Figure 8A, B). Occasionally, we also observed “packets” of sperm within the oviduct en route to the seminal receptacle (data not shown). From the seminal receptacle sperm are able to begin the fertilization process as oocytes emerge from the ovary. Oocytes then pass anteriorly though the oviduct (Figure 8A), which merges with the vitelline duct (now the ovo-vitelline duct), before reaching a mass of secretory cells collectively referred to as Mehlis' gland. A variety of functions have been proposed for Mehlis' gland, including providing lubrication for the reproductive tract, activating sperm, and providing aterials for egg shell biosynthesis [53], [54]. Similar to histological [51] and ultrastructural descriptions [55], [56], we observe this gland directly posterior to the ootype (Figure 8C). This observation contrasts that of Neves et al. (2005) [18] who describe the columnar epithelium of the ootype (described by Erasmus [55] and Gönnert [51]) as Mehlis' gland. We found the projections of Mehils' gland cells to label with both sWGA and PNA (Figure 8C, D and Movie S6). Furthermore, we observed anti-acetylated α-tubulin to label microtubules (described previously by Erasmus [55]) at the distal projections of these cells before they enter the ootype (Figure 8C and Movie S6). In cross section the cells of Mehlis' Gland labeled with sWGA, PNA, and anti-acetylated α-tubulin can be seen projecting radially into the posterior ootype (Movie S6). Within the ootype the eggshell coalesces to surround a single fertilized egg and 30–40 vitelline cells [57] (Shown in Figure 8C and Movie S6). This egg is passed anteriorly through the uterus and out the female genital pore.

**Figure 9:**
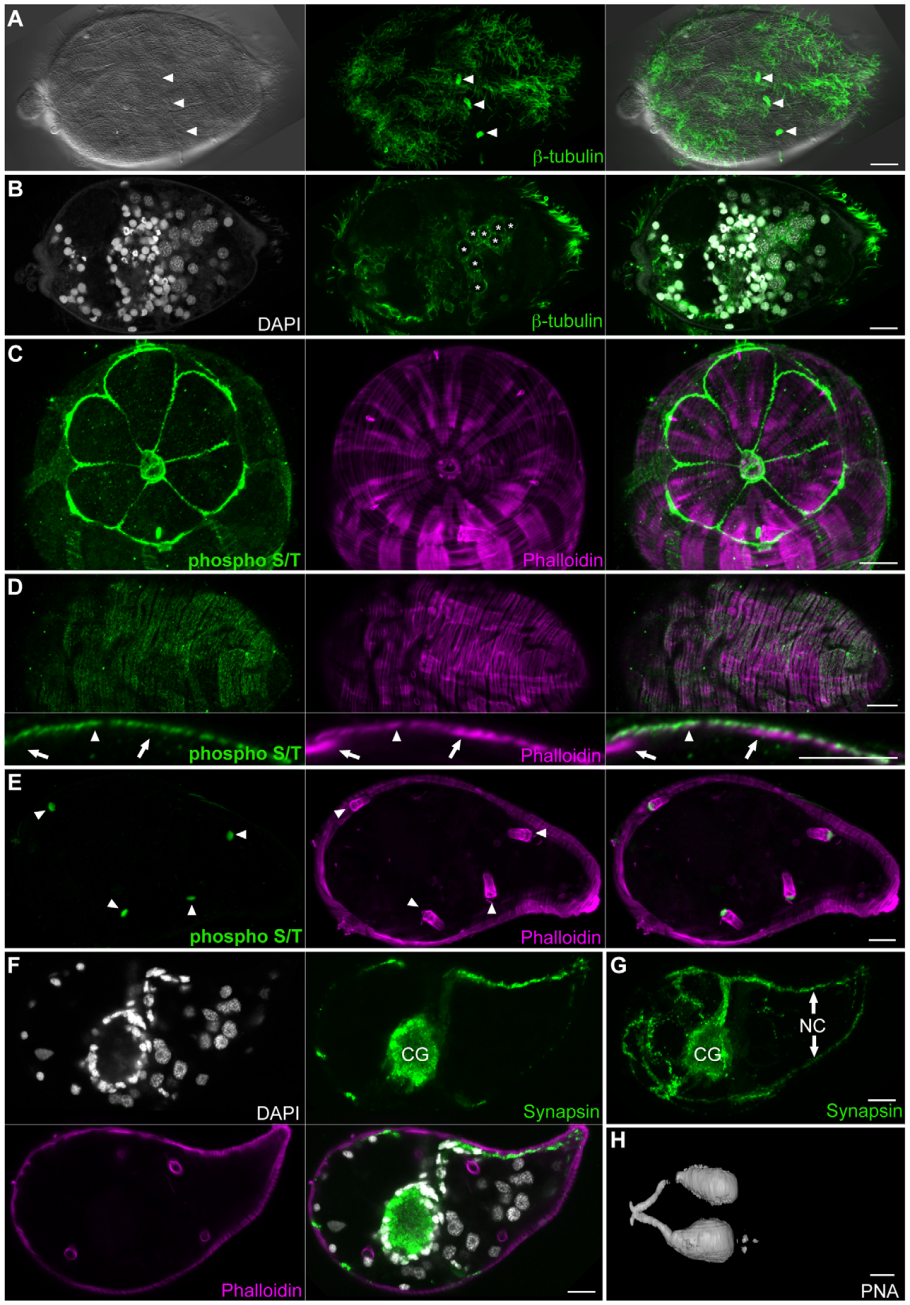
The organ systems of miracidia. (A) The miracidial surface is covered by motile cilia on the epidermal plates as well as multiciliated sensory papilla (arrowheads) visualized by staining for β-tubulin and DIC optics. (B) Mid-level confocal section showing microtubule meshwork of germ cells (asterisks). (C) Anti-phospho S/T strongly labels the terebratorium and epidermal ridges surrounding the first tier of epidermal plates. (D) Anti-phospho S/T also displayed a weaker circumferential banding pattern similar to circumferential muscle (top). Optical crossections indicate that the weak, superficial anti-phospho S/T labeling is at the level of circumferential muscles (arrowhead) but not longitudinal muscles (arrows). (E) Similar to cercariae, anti-phospho S/T strongly labels the base of flame cells (arrowheads) in miracidia. (F and G) Immunofluorescence with an anti-synapsin antibody labels the cephalic ganglia and peripheral nerve structures. (F) Mid-level confocal section showing labeling of neuropil of the cephalic ganglia with anti-synapsin (green). The neuronal cell bodies of the cephalic ganglia contain small nuclei that stain intensely with DAPI (grey) and surround the neuropil. Phalloidin staining (magenta) labels the muscle as well as flame cells. (G) Maximum intensity projection of a miracidium stained with anti-synapsin antibody. Abbreviations: Cephalic ganglia (CG), nerve cords (NC). (H) Volume rendering of miracidium stained with PNA showing labeling of lateral glands and ducts. Scale Bars, 10 µm. Anterior faces left in all panels except C that represents a view from the anterior surface.

Upon release into freshwater, eggs generated by the adult schistosome hatch, giving rise to free-living miracidia. Following hatching, miracidia must locate and infect an appropriate snail host. Like the cercariae, miracidia contain a number of ciliated sensory structures on their surface [58]; these can also be labeled with anti-tubulin antibodies (Figure 9A, arrowheads). In addition to labeling the multiciliated sensory papilla, anti-tubulin antibodies also label the numerous motile cilia of the epidermal plates that cover the animal. Interestingly, anti-β-tubulin also showed strong labeling of a cytoplasmic meshwork of microtubules in the germ cells (Figure 9B). Previous ultrastructural analysis of miracidial germ cells suggest that these cells may be anchored through several small projections that extend into the intercellular space [58]. It is possible that the microtubule meshwork we observed functions to generate or maintain these cellular projections.

Phalloidin staining of miracidia facilitates analysis of the elaborate body wall musculature and flame cells (Figure 9C–E, and Movie S7). A detailed description of phalloidin staining has previously been reported, and we refer readers to that work [59]. In cercariae we observed anti-phospho S/T staining of the longitudinal muscles in the tail (Figure 1I). Similarly we observed anti-phospho S/T staining in the miracidia in a circumferential banding pattern similar to that observed for phalloidin staining of the muscle (Figure 9D). It was difficult to ascertain whether this labeling was in the muscle layer itself or in the overlying epidermal plates and ridges. In addition to the superficial banding pattern, we observed anti-phospho S/T labeling of the base of flame cells (Figure 9E bottom arrowheads).

The central nervous system of miracidia resembles that observed in cercariae. The neural mass is centrally located, stains with the anti-synapsin antibody, and is surrounded by neuronal cell bodies that are identifiable by their small ovoid nuclei which stain intensely with DAPI (Figure 9F and Movie S7). Several nerve cords project anteriorly while only a dorsal and ventral nerve cord project posteriorly (Figure 9F, G).

Miracidia contain three glands, an apical gland and two lateral glands [58]. Although we observed staining of the lateral glands with a number of the lectins (Figure 9H and Table S1), only a few lectins were observed staining the apical gland (Table S1). This suggests that the products of the apical gland are likely different than those of the lateral glands, an observation consistent with ultrastructural studies demonstrating differences in size of secretory vesicles in these glands [58]. While several lectins stained ECM and other non-secretory cells in cercariae and adults, we failed to observe significant staining of other cell types in miracidia. While this may reflect real biological differences in miracidial tissues compared to cercarial or adult tissues, it is also possible that our fixation protocol for miracidia either blocked epitopes or failed to fix them sufficiently.

## Discussion

These studies describe a number of robust cell-specific labels for three life-cycle stages of *S. mansoni*. One interesting observation from these studies was that a diverse collection of fluorescently labeled lectins stained secretory glands in all life-cycle stages observed. Specifically, lectins stained the acetabular and head glands of cercariae, the esophageal and Mehlis' gland of adults, and the apical and lateral glands of miracidia. These observations are consistent with previous studies describing lectins, including PNA and WGA, labeling glands, or their secretions [31], in live miracidia and cercariae [34]. Because many of these same lectins label secretory cell types in planarians [29], it is possible these types of cells share a common evolutionary origin and their functions have diverged to accommodate the free-living or parasitic lifestyles of these animals.

Many of the reagents we examined labeled portions of the protonephridial system at a number of life-cycle stages. While one of the major functions ascribed to protonephridia is osmoregulation, the system is not only maintained but becomes elaborated in adults when the parasite conforms to the osmolarity of its host [44]. Recent studies demonstrating uptake of molecules including fluorescent analogs of Pgp/MRP substrates and a fluorescent derivative of praziquantel [60], [61], [62], [63] suggests that the protonephridial system is likely important in drug metabolism and excretion. The combination of morphological markers we have identified, along with fluorescent dye labeling experiments, will be instrumental in analyzing protonephridial phenotypes in future drug and RNA interference studies.

Since egg-induced granuloma formation is the principle cause of the pathology of schistosomiasis, understanding reproductive processes in schistosomes could illuminate new therapeutic opportunities. Using lectins, an antibody against acetylated α-tubulin, and two common fluorescent stains (DAPI and phalloidin, which has been described previously [21]) we were able to clearly visualize the gonads (testes and ovaries) and most accessory reproductive structures (e.g. ootype and Mehlis' gland) in adult schistosomes. The combination of these reagents provides a step forward from more general fluorescence-based approaches previously used to describe the schistosome reproductive system (e.g. carmine red staining [18]), and should provide a useful tool for accessing reproductive development in these parasites.

Collectively, these studies show the utility of this approach for identifying robust markers of schistosome tissues. We anticipate that similar studies focused on other life-cycle stages (e.g. schistosomula and sporocysts) will yield comparable results and will provide additional tools that will enable researchers to dissect the many fascinating biological questions raised by these important parasites.

## Supporting Information

Movie S1 **Phalloidin staining of** ***S. mansoni*** **cercariae.** Shown are Z-stacks through the head of a cercariae stained with phalloidin. DIC optics and DAPI staining are also shown overlaid with phalloidin staining.
(MP4)

Movie S2 **Pre- and post-acetabular glands.** First, Z-stacks though the head of a cercariae labeled with lectins PNA and PSA. Overlay with phalloidin staining is also shown. Second, 3D rendering showing the distal projections of the acetabular glands at the anterior of the animal.
(MP4)

Movie S3 **Whole cercaria view of anti-phospho Y immunofluorescence.** First, a Z-stack showing immunofluorescence with the anti-phospho Y antibody. Second, a 3D rendering of anti-phospho Y immunostaining.
(MP4)

Movie S4 **Visualization of the cercariae CNS with anti-synapsin.** First, Z-stacks showing immunofluorescence staining with anti-synapsin in a cercarial head. Overlay with DAPI, DIC and phalloidin are also shown. Second, 3D renderings of anti-synapsin immunostaining.
(MP4)

Movie S5 **The protonephridial system of adult** ***S. mansoni*** **.** First, 360° rotation of a 3D rendering with depth coding showing ciliated regions of the protonephridial system visualized by immunostaining with anti-acetylated α-tubulin. In this projection warmer colors (e.g. reds) represent more superficial structures, whereas cooler colors (e.g. blues) correspond to deeper structures. Second, a 360° rotation of a protonephridial unit and its associated ducts visualized by staining with sWGA and anti-acetylated α-tubulin.
(MP4)

Movie S6 **The ootype/Mehlis' gland complex.** First, Z-stacks showing various regions of the ootype and Mehlis' gland visualized with DIC optics and DAPI, PNA, sWGA, and anti-acetylated α-tubulin stainings. Second, a movie of optically derived cross-sections through Mehlis' gland and the ootype. Sections are shown moving from the posterior end of Mehlis' gland towards the anterior of the ootype.
(MP4)

Movie S7 **3D renderings of miracidia.** 3D renderings of miracidia stained with phalloidin, anti-synapsin and DAPI.
(MP4)

Table S1 **Reagents used in this study and their labeling patterns in *Schistosoma mansoni.***
(PDF)
