# Supplementary material for: An Atlas for Schistosoma mansoni Organs and Life-Cycle Stages Using Cell Type-Specific Markers and Confocal Microscopy
Source: PLoS Negl Trop Dis. 2011 Mar 8;5(3):e1009. doi: 10.1371/journal.pntd.0001009 (PMC3050934; doi:10.1371/journal.pntd.0001009)
Supplement: Table S1 — Reagents used in this study and their labeling patterns in Schistosoma mansoni. (PDF) [file pntd.0001009.s008.pdf]

Table S1. Reagents used in this study and their labeling patterns in *Schistosoma mansoni.*

Reagent	Reagent Type	Source	Dilution from stock	Proteinase K treatment	Cercariae	Miracidia	Adults
Anti-acetylated α-tubulin (Clone 6-11B-1)	Mouse Mono-clonal antibody	Santa Cruz	1:500	+	Sensory cilia, flame cells, microtubular network of acetabular glands	Flame cells, cilia of epidermal plates, sensory cilia	Flame cells, protonephridial collecting ducts, sperm
Anti-β-tubulin (Clone E7)	Mouse Mono-clonal antibody	Developmental studies hybridoma bank	1:500	+ or -	Sensory Cilia*, neural projections of tail*, flame cells and ciliated protonephridial ducts	Flame cells, cilia of epidermal plates*, Sensory cilia*, microtubular network of germballs*	ND
Anti-ε-tubulin (Clone TUB-11)	Mouse Mono-clonal antibody	Sigma	1:500	+	weak CNS (neuropil), sensory cilia, flame cells, subset of nuclei in tail	ND	ND
Anti-phospho S/T	Mixed mouse Monoclonal antibodies	Upstate (Cat. No. 44-006)	1:500	-	Longitudinal muscles of tail, weak CNS (neuropil of cephalic ganglia), flame cell (ciliary rootlets), most nuclei (not germ cell precursors)	Weak muscle, most nuclei, flame cell (ciliary rootlets),	ND
Anti-phospho Y (P-Tyr-100)	Mouse Mono-clonal antibody	Cell Signaling	1:500	+	moderate CNS (neuropil of cephalic ganglia and cords), flame cells, protonephridial duct in tail, acetabular glands	ND	ND
Anti-Synapsin (Clone 3C11)	Mouse Mono-clonal antibody	Developmental studies hybridoma bank	1:50	-	CNS (Neuropil of cephalic ganglia and cords)	CNS (neuropil of cephalic ganglia and cords)	ND
Erythina chistagalli lectin (ECL)	Lectin	Vector Laboratories	1:500	+/-	Post-acetabular glands/ducts (periphery), pre-acetabular glands (weak, blotchy)	Lateral glands, weak anterior gland	ND
Griffonia simplicifolia lectin I (GSLI)	Lectin	Vector Laboratories	1:500	+/-	Post-acetabular glands/ducts (periphery), pre-acetabular glands (weak)	Weak lateral glands	ND
Griffonia simplicifolia lectin II (GSLII)	Lectin	Vector Laboratories	1:500	-	Post-acetabular glands/ducts (periphery), head gland, protonephridial duct	Lateral glands	ND
Jacalin	Lectin	Vector Laboratories	1:500	-	Pre- and post-acetabular glands, flame cell caps, weak protonephridial ducts	Lateral glands	ND
Lens culinaris lectin (LCA)	Lectin	Vector Laboratories	1:500	+/-	Pre- and post-acetabular glands/ducts, ECM (e.g. basement membrane, esophagus, flame cell)	Weak lateral glands	ND
Lycopersicon esuleatum lectin (LEL)	Lectin	Vector Laboratories	1:500	+/-	Post-acetabular glands/ducts, flame cell, protonephridial ducts ECM/BM	Lateral glands	ND
Peanut Agglutinin (PNA)	Lectin	Vector Laboratories	1:500	+	Glycocalyx/tegument, post-acetabular, glands/ducts (periphery)<br>Pre-acetabular glands/ducts, ECM of flame cell	Lateral glands	Mehlis' Gland, esophageal gland, vitellaria, weak basement membrane, weak labeling at base of flame cell
Pisum sativum Agglutinin (PSA)	Lectin	Vector Laboratories	1:500	+/-	ECM (e.g. basement membrane, esophagus, flame cell), post-acetabular glands/ducts (central), neuropil	Weak lateral gland	ND
Ricinus communis Agglutinin I (RCA)	Lectin	Vector Laboratories	1:500	+/-	Post-acetabular glands/ducts (periphery), Pre-acetabular glands	Weak lateral gland	ND
Soybean Agglutinin (SBA)	Lectin	Vector Laboratories	1:500	+/-	Post-acetabular glands/ducts, pre-acetabular glands	Lateral glands	ND
Sophora japonica Agglutinin (SJA)	Lectin	Vector Laboratories	1:500	+/-	Post-acetabular glands/ducts, weak pre-acetabular glands	Weak lateral glands	ND

Solanum tuberosum Lectin (STL)	Lectin	Vector Laboratories	1:500	+/-	Post-acetabular glands, weak pre-acetabular glands, head gland, weak basement membrane	Weak lateral glands	ND
Succinylated Wheat Germ Agglutinin (sWGA)	Lectin	Vector Laboratories	1:500	+	Post-acetabular glands/ducts, pre-acetabular glands, glycocalyx/tegument, neuropil, protonephridial ducts, head gland	Lateral glands	Unciliated (strong) and ciliated (weak) protonephridial ducts, ECM of many cells, ootype, male tubercles, basement membrane, brain neuropil
Ulex europaeus Agglutinin I (UEA I)	Lectin	Vector Laboratories	1:500	+/-	Tegument between actin spines, post-acetabular glands/ducts, weak pre-acetabular glands, head gland	Weak lateral glands	ND
Vicia villosa Lectin (VVL)	Lectin	Vector Laboratories	1:500	+/-	Post-acetabular Glands	Lateral glands, weak anterior gland	ND
Wheat Germ Agglutinin (WGA)	Lectin	Vector Laboratories	1:500	+	Post-acetabular glands/ducts, weak pre-acetabular glands, basement membrane, neuropil, head gland	Lateral glands	ND

*Indicates which staining patterns were specifically observed when Proteinase K was omitted.
